# Causal Cognition, Force Dynamics and Early Hunting Technologies

**DOI:** 10.3389/fpsyg.2018.00087

**Published:** 2018-02-12

**Authors:** Peter Gärdenfors, Marlize Lombard

**Affiliations:** ^1^Cognitive Science, Department of Philosophy, Lund University, Lund, Sweden; ^2^Stellenbosch Institute for Advanced Study, Wallenberg Research Centre, Stellenbosch University, Stellenbosch, South Africa; ^3^Centre for Anthropological Research, Department of Anthropology and Development Studies, University of Johannesburg, Johannesburg, South Africa

**Keywords:** causal cognition, cognitive evolution, force dynamics, hunting technology, spears, bow and arrow

## Abstract

With this contribution we analyze ancient hunting technologies as one way to explore the development of causal cognition in the hominin lineage. Building on earlier work, we separate seven grades of causal thinking. By looking at variations in force dynamics as a central element in causal cognition, we analyze the thinking required for different hunting technologies such as stabbing spears, throwing spears, launching atlatl darts, shooting arrows with a bow, and the use of poisoned arrows. Our interpretation demonstrates that there is an interplay between the extension of human body through technology and expanding our cognitive abilities to reason about causes. It adds content and dimension to the trend of including embodied cognition in evolutionary studies and in the interpretation of the archeological record. Our method could explain variation in technology sets between archaic and modern human groups.

## Introduction

We argue that an increased capacity to reason about causes enabled a development in tool technology along the hominin line. The main cognitive development behind the more advanced form of causal cognition is the unique hominin ability to mentally represent force dynamics. We illustrate the expansion of reasoning about forces by a study of different kinds of hunting technologies (stabbing spears, throwing spears, launching atlatl darts, shooting arrows with a bow, and the use of poisoned arrows) and what types of casual reasoning they involve.

Hominin hunting technologies represent a class of material culture that is often centered on ‘force.’ Some hunting technologies can be traced through time (e.g., Lombard, 2011; Sisk and Shea, 2011; Yaroshevich et al., 2016), providing a window on the evolution of applied force dynamics. Tools used during non-human primate hunting provide insight into their material engagement during some hunting activities (e.g., Preutz and Bertolani, 2007). Malafouris (2013, p. 7) stresses that “understanding the relationship between cognition and material culture what it is, how it changes, and what role the human body plays in forging those links is of the utmost importance for the study of mind.” Today, humans are the only group who has been, or can be defined as, a species based on our relationship with technology (e.g., Ambrose, 2001; Boivin, 2008; Barham, 2013; Lombard, 2016), so that tracing the development of such a relationship may throw valuable light on how we became who/what we are. We extend our bodies, and by implication our minds, through the ‘prosthetic’ use of technology, and such extension can go beyond physical reach (Malafouris, 2013), exerting ‘remote control’ over our socio-economic environments.

In the recent ‘third hand’ debate Bruner and Lozano (2014) also argue that without technology it would be impossible for the human mind to do what is does or to be what it is. They draw on ‘extended mind’ theory in which our cognition and neural circuits are profoundly rooted in and shaped by object engagement. Neurologically, objects are interpreted differently when they are within body range (i.e., if they can interact physically with the body, occupying a peri-personal area instead), as opposed to being without reach (extra-personal space materiality) (e.g., Berti and Frassinetti, 2000; Maravita et al., 2003; Maravita and Iriki, 2004; Davoli et al., 2012). In short, based on attributes of Neanderthal teeth, it was argued that *Homo neanderthalensis* and probably *H. heidelbergensis* used their mouths as a third hand “because of an incomplete brain-environment body interface and limits in visuospatial integration ability” (Bruner and Lozano, 2014, p. 278). This suggests that the Neanderthals had a limited ability to conceptualize and use technologies that function across distance and time. Bruner and Lozano (2014) aimed to provide an alternative interpretation as opposed to the bio-cultural information gained from paleontology and archeology, but acknowledged that their conclusion is difficult to test. We suggest that by looking at force dynamics through the lens of causal cognition and hunting technologies, it is possible to further enrich our repertoire for thinking about variability in hominin cognitive evolution.

## Seven Grades of Causal Cognition

Our first task is to delineate the notion of causal cognition. Unlike many philosophers who view causation as an aspect of the world, we aim for a psychological account of how humans, including our ancestors, and non-human animals thought/think about causes. We do not suppose that there is only one form of causal thinking. In this respect we agree with Woodward (2011), who identified three kinds of learning about causal relations [see also Gärdenfors (2003), section 2.8, for a related account]. He calls the first kind ‘egocentric learning’ and defines it as the ability to learn that one’s own physical actions can cause certain outcomes. This form of learning is based on ordinary operant or instrumental conditioning and allows an individual to perceive itself as an agent, that is, to be aware that by controlling one’s body one can control certain aspects of the environment and one’s relation to it.

Woodward’s second kind is ‘agent causal learning,’ where an individual also learns about causes from the actions of others. This form of causal thinking involves understanding that the outcomes of the actions of others have implications for what would result from an individual’s own actions. For example, a young chimpanzee that observes her mother cracking a nut by using a hammer stone can grasp that she too should be able to achieve a similar result by following similar actions. Being able to learn causal relations from the actions of others makes it possible for the individual to imitate/emulate behaviors (Tomasello, 1999). It sometimes also involves certain forms of mindreading, that is, understanding what goal and desires the other individual have.

The third kind Woodward calls ‘observation/action causal learning.’ This involves being able to integrate a range of natural signs or patterns with the egocentric and agent causal reasoning. We argue in section “The Role of Force Dynamics in Causal Cognition” that this form of learning requires mental representations of the different kinds of forces underlying the causal interactions. Woodward (2011, p. 38, 39) notes that the available empirical evidence suggests that apes are not observation/action causal learners (also see Tomasello and Call, 1997).

Even though Woodward does not write about the kinds of causal learning in evolutionary terms, it can be argued that since many animal species are egocentric causal learners, but not agent causal learners, his three kinds represent a form of cognitive development. Our use of the term grade implies that the different kinds of causal understanding we describe probably phased into each other throughout our evolution, but does not imply that we subscribe to a *scala naturae* with human cognition as the pinnacle (see Lombard and Gärdenfors, 2017). Nevertheless, Woodward’s three types and our expansion into seven grades involve increasing forms of understanding of actions, forces and the minds of others. We believe this motivates our choice of terminology.

Building from Woodward (2011), Lombard and Gärdenfors (2017) propose a finer gradation of the emergence of causal cognition in humans. We briefly present the seven grades of that model (for detailed discussion see Lombard and Gärdenfors, 2017):

### Grade 1: Individual Causal Understanding

The first grade involves a direct connection between a motor action that an individual executes and the resulting effect. Typical examples are a baby kicking its foot, learning the connection between motor commands and the resulting actions, or a kitten playing with a toy. In this case both the cause and the effect are directly perceived. The result is that the individual experiences its own agency. This grade need not involve strong cognitive mechanisms, but can be explained via learning by conditioning.

The first grade corresponds to Woodward’s (2011) egocentric causal learner. The following grades 2–5 represent a partitioning of his agent causal learners.

### Grade 2: Cued Dyadic-Causal Understanding

This grade involves two individuals who take turns in performing a similar action. One example is two puppies rough-and-tumble playing, taking turns in attacking (Bekoff and Byers, 1998). The motor forces behind the other individual’s actions are not directly perceived, but they are inferred via a mapping onto the forces involved in one’s own actions. Thus, I understand that the action of the other causes an effect because it gives the same result as my own action. On this grade, one individual understands the agency of the other.

### Grade 3: Conspecific Mindreading

Humans understand how our desires, intentions and beliefs lead to different kinds of actions. One example of detached dual-causal understanding is gaze following, i.e., understanding that if someone is looking firmly in a particular direction, there is something worthy of attention in that direction. In other words, the onlooker infers that there is a cause for the gaze direction, even if the onlooker itself cannot perceive the cause, but uses his or her understanding of the inner state of the one looking as a cause of the behavior. On this grade, attention, desires, intentions and beliefs are seen as ‘mental forces’ causing the action (see Gärdenfors, 2003, 2007).

### Grade 4: Detached Dyadic-Causal Understanding

Sometimes we do not perceive the actions of somebody else, but only the traces of them. An example is the tracks of a person in the snow leading up to your house. The cause is detached from the present situation (Gärdenfors, 1995). I don’t see the person, but conclude that somebody’s presence in the past is the cause of the tracks. I infer the agency of somebody else, which leads to the presence of the tracks. This grade depends on the capacity to entertain two mental representations at the same time, that is, the current perceptual state of seeing the tracks together with my imagination of the person being present in the field.

Several experiments and observations indicate that monkeys and apes often do not infer physical causes from their effects (e.g., Cheney and Seyfarth, 1990; Povinelli, 2000, but see Mulcahy and Call, 2006), hence they do not reach this grade. Being able to reason from effects to *non-present* causes seems to be unique to humans. In line with this, Tomasello and Call (1997) suggest that apes are not observation/action causal learners in the sense of Woodward (2011).

### Grade 5: Causal Understanding and Mindreading of Non-conspecifics

We sometimes have a dyadic-causal understanding of the actions and intentions of other species, although their motor actions and cognitive processes are different from ours. The most interesting case in relation to hunting is human detached causal understanding of non-human animals. For example, when I see an animal track, I can sometimes infer the cause, since I recognize the track as that of a gemsbok. With increased experience, I may also be able to infer the mental states of the animal, for example, if I see blood in the tracks and that the gemsbok is limping, I can, first, draw the conclusion that the gemsbok is hurt by using detached dyadic-causal reasoning, and, second, that it is in pain by using a form of empathy mirroring my own experiences of being hurt. The difference, between grades 3 and 4 on the one hand, and grade 5 on the other, is gradual and depends to a large extent on the experience of the behavior of other species. The actions and mental states of other animals map less directly onto our own, since their bodies and their inner worlds are different, but we can learn the mapping.

Our main reason for making the distinctions between grades 2–5 is that they involve increasing use of detached representations and of mindreading. We argue later that the detached representations are necessary for the historically later forms of hunting technologies.

Our two last grades involve a partitioning of Woodward’s (2011) observation/action causal learner.

### Grade 6: Inanimate Causal Understanding

We reach a more advanced grade of causal understanding when we can ascribe causal roles to inanimate objects. I see a twig being stuck in the resin on the trunk of a tree or I see an animal being stuck in the mud of the drying waterhole. Again I don’t perceive the cause, but I infer the forces from the resin or the mud. Such understanding would enable me, to make a direct correlation between the use of resin or tree gum as an adhesive, and enable me to construct a composite tool such as a stone-tipped spear for hunting. Unlike the previous cases, there is no animate agent that performs an action. For this grade we argue in the following section that causation is understood in terms of force dynamics (Povinelli, 2000; Wolff, 2007; Gärdenfors and Warglien, 2012) as an extension of agency.

### Grade 7: Causal Network Understanding

We suggest that the most complex grade of causal cognition is the understanding of how domain-specific causal node sets connect or link to inter-domain causal networks (e.g., Tenenbaum and Niyogi, 2003). The most advanced form of this kind of reasoning is science (i.e., hypothetical reasoning) (e.g., Gopnik et al., 1999). Thus, with causal network understanding I am able to abstract the knowledge gained from one domain and apply it to another by imagining how past scenarios can be used in the future to solve a range of unrelated problems innovatively. For example, if by understanding the interplay between forces and counterforces I have learned how to fasten a stone tip to a spear (grade 6 causal understanding), now equipped with grade 7 causal understanding, I also understand that the abstract principle of fastening one object to another can be used in an endless array of disconnected contexts. Such understanding could help me to engineer a snare for catching prey or to combine several different materials to construct a shelter or vessel to cross bodies of water, etc. (for further discussion and examples see Lombard and Gärdenfors, 2017).

During this grade of causal understanding, aspects of all the previous causal understanding grades can be integrated and mapped onto each other into increasing complexity.

### Further to Our Model

The theoretical framework of our 7-grade model for the evolution of causal cognition is based on the levels of detachment from egocentric learning or individual understanding. Compared to Woodward’s (2011) classification, we provide a more nuanced understanding of the evolution of causal cognition, which is testable against empirical data from the palaeoanthropological and archeological records (e.g., Lombard and Gärdenfors, 2017). In particular, by splitting Woodward’s agent causal learners into grades 2–5, we have previously concluded that non-human animals manage grade 2, do it less well than humans on grade 3 and are very limited when it comes to grade 4 (Lombard and Gärdenfors, 2017). It is key, however, to appreciate that even though the simpler grades of causal understanding support or scaffold the more complex ones, our framework does not automatically denote a unilinear evolutionary trajectory (e.g., Haidle et al., 2015; Lombard, 2016). Within each grade of causal understanding there might be several levels of complexity that developed at different times in different places and/or in different hominin populations (Lombard and Gärdenfors, 2017).

## The Role of Force Dynamics in Causal Cognition

Our main hypothesis concerning the difference between human causal cognition and that of other animals is that non-human animals understand causation only in terms of agency (Woodward’s first two kinds, our grades 1–5), while humans can reason about causes also via forces that operate across space (action at a distance) and through time [decoupling in the sense of Hockett (1960) or detachment in the sense of Gärdenfors (1995, 2003)]. In the following section we want to connect such reasoning about forces with different forms of hunting technologies. Before we do this we need to present the role of forces in causal thinking in greater detail.

Monkeys are surprisingly restricted in their reasoning about physical causes of phenomena, as has been shown by Povinelli (2000) and others. In contrast, even small human children show strong signs of interpreting the world with the aid of hidden forces and other causal variables. Gopnik (1998, p. 104) claims that “other animals primarily understand causality in terms of the effects of their own actions on the world. In contrast, human beings combine that understanding with a view that equates the causal power of their own actions and those of objects independent of them.” Going from being an agent causal learner to being an observation/action causal learner involves a shift in focus from actions to the underlying forces (Gärdenfors and Warglien, 2012; Gärdenfors, 2014). The transition is difficult as is witnessed by children’s tendency to animistic reasoning. This tendency indicates that inanimate causal understanding takes time to achieve in human development.

Woodward (2011, p. 28) argues that ‘force transmission’ is not sufficient to explain causal reasoning. In the psychological literature, force transmission has typically been interpreted as transmission via physical contact (e.g., Leslie, 1995). It is important to note that our use of force dynamics is much broader. Human perception of physical forces is presumably primary, but in interaction between humans it has been extended to include emotional and social forces in situations involving threats, promises, persuasions, seduction, etc. Furthermore, early on in history, humans have learned to utilize the ‘forces’ that are stored in various objects: medicine or poison in plants, heat in firewood, etc. (e.g., Wadley, 2013). Within grade 6 of causal reasoning, one can speculate about a development from direct force transmission, to transmission at a distance in space and then to stored physical forces (transmission at a distance in time), as in spring traps and bows, and finally to non-physical ‘forces.’

In Lombard and Gärdenfors (2017) we applied the seven-grade model to the evolution of tracking as an example. In this article we analyze the causal cognition involved in early forms of tool use, in particular tools for hunting. We focus on inanimate causation, that is, grades 6 and 7, since it is the understanding of the causal effects of the hunting tools that will be relevant.

## Force Dynamics in Hunting Technologies

Our thesis is that an expanding understanding of physical force dynamics is necessary for the construction and use of increasingly sophisticated hunting tools. It may even be that the fitness advantages of the emerging technologies have functioned as a selective pressure for increasing causal reasoning concerning physical forces (see also Wolpert, 2003; Malafouris, 2013).

### Tools as Force Extensions

From an evolutionary point of view an interesting question is what have been the selective mechanisms behind the human capacity for inanimate causal reasoning. As mentioned in the previous section, tool use or technology may have played an important role. Tools extend your peri-personal space – they allow you to act at a distance and to alter the force patterns generated by your body. When you hit a nut with a stone you magnify the forces acting on the nut compared to pounding on the nut with your hand and when you poke with a stick into a hole you extend the poking abilities of your fingers. Such tool use represents basic causal understanding that can be roughly associated with grades 1 and 2. With this as a background for the development of hunting technologies, we now turn to an analysis of the different forms of causal reasoning involved in the technologies.

### Directly Acting Forces

Some hunting weapons are based on thrusting, which represents an understanding of direct force transmission. For example, a thrusting spear never leaves the hand of the hunter, it simply functions as an extension of the arm modifying the forces exerted. Weapon engagement with the target is immediate, thus there is only a short physical distance between hunter and prey. There is no time dimension (delayed contact) involved.

We see that when chimpanzees hunt bush babies with sharpened sticks, they thrust at their prey (e.g., Preutz and Bertolani, 2007), they do not hunt by throwing. Although they throw objects occasionally, currently only humans habitually ‘throw projectiles with high speed and great accuracy’ at targets at a distance (Roach et al., 2013). Thrusting does not need the powerful thumb-tip to finger-tip prehension associated with humans (e.g., Young, 2003, 2009; Roach et al., 2012), nor a specialized ‘throwing’ shoulder (e.g., Shaw and Stock, 2009; Roach et al., 2013; Larson, 2015). In both cases, the physical traits associated with ‘pinching’ and ‘throwing’ evolved subsequent to ‘grasping’ and ‘thrusting’ in the hominin record. We therefore argue that the cognitive traits for thrusting also evolved earlier than for habitual, accurate and forceful throwing. Because force transmission from arm to spear to target is direct, and the thrusting effect is felt directly in the arm, there is a strong mapping between cause and effect so that thrusting does not require any of the advanced forms of causal understanding.

When sharp stone flakes or points are hafted to wooden shafts (an element of grade 6 causal understanding), the force dynamics of spears are changed. With such composite weaponry a cutting or slicing force is added to that of thrusting enhancing a hunter’s ability to harm prey effectively. Understanding to make stone-tipped weaponry would thus have adaptive value, and from the archeological record it is clear that *Homo heidelbergensis*, the Neanderthals and *H. sapiens* hunted with stone-tipped spears (Lombard, 2005; Villa and Soriano, 2010; Wilkins et al., 2012; Haidle et al., 2015).

Technologically and cognitively, simple wooden spears are generally thought to precede stone-tipped ones (e.g., Haidle, 2010; Lombard and Haidle, 2012; Haidle et al., 2015). It is generally accepted that stone-tipped spears were habitually used for hunting from about 300 thousand years ago (McBrearty and Tryon, 2006; Lombard, 2012). Direct evidence for the use of wooden spears date to about 400 thousand years ago (Dennell, 1997).

### Forces Acting at a Distance

Even further extensions of your peri-personal space are achieved when the tool leaves the direct control of your body and exerts its force at a distance; i.e., becomes extra-personal space. Throwing an object like a stone or a stick may be the first method of force transmission at a distance (see Calvin, 1993 for a speculative account). Chimpanzees and other apes and monkeys throw branches and rocks, mainly as a way of intimidating predators or rivaling conspecifics. Their ability to aim is limited (e.g., Westergaard and Suomi, 1993; Westergaard et al., 2000; Roach and Lieberman, 2014). During the evolution of the hominins, not only the shape of the hand but also the shoulder and humerus changed in such a way that made throwing much more effective both in terms of strength and in terms of aiming accuracy (Roach et al., 2013). Throwing spears represent the indirect transmission of arm force. The thrusting effect of the spear is detached in *space* from the thrower. This entails that the mapping between cause and effect must be inferred from the behavior of the animal that is hit. In learning such mapping, some representation of force transmission and therefore a causal reasoning of grade 6 is required.

It is reasonable to argue that, as an extension of the initial intimidation posturing, the ability to throw with force and accurately hit a target became selectively advantageous for hominins who had to defend themselves, their offspring and food sources from dangerous predators and scavengers on the African landscape (e.g., Lombard, 2015). Acting at a distance also brings greater safety for the thrower, whether in hunting or defense, since it reduces risk of injury from contact with the prey or the enemy (e.g., Villa and Soriano, 2010).

The anatomical adaptations that enable elastic energy storage and release at the shoulder first appear in their ‘modern’ configuration in *H. erectus* 2 million years ago (e.g., Roach et al., 2013). We do not necessarily see this as evidence of hunting (there is no unambiguous evidence for hunting at this time), but more parsimoniously as part of high-level scavenging behavior. For example, it would have been of great advantage to be able to throw objects and hit competing scavengers to gain safe access to carcasses. This would imply, however, that the roots of inanimate causal understanding (grade 6) are relatively old, and that by the time we see the earliest evidence for spear hunting, it was present at least in rudimentary form to be further developed. This is in line with previous interpretations by cognitive archeologists who argue that *H. heidelbergensis* (the ancestor to both the Neanderthals and *H. sapiens*) were proficient spear hunters (e.g., Thieme, 2007; Lombard and Haidle, 2012; Conard et al., 2015).

A high level of inanimate causal understanding is probably represented by atlatl (spear-thrower and dart technologies), where the atlatl becomes a further force extension that increases arm leverage and the distance between hunter and prey (e.g., Palter, 1977; Butler, 1975; Villa and Soriano, 2010). Brooks et al. (2006) have argued that atlatls could have been used in southern Africa by about 100 thousand years ago. We therefore suggest that the ability to throw objects with force and accuracy, using increasingly complex technologies to do so, represent phases in the cognitive evolution of grade 6 causal understanding.

### Stored Forces

Technologies involving bow and arrow or snares (that use bent branches that are released when the snare is touched) involve causal reasoning concerning indirect transmission of force via stored energy. This is in contrast with using basic leverage for spear throwing and increased leverage obtained through the use of technologies such as the atlatl. Bow hunting probably have been in use from as early as about 70–64 thousand years ago in Africa (Backwell et al., 2008; Lombard and Phillipson, 2010; Brown et al., 2012). Although the atlatl represents grade 6 of causal cognition as well as a mechanically aided delivery system (e.g., Lombard and Haidle, 2012), it does not represent causal network understanding, that is grade 7. As Butler (1975, p. 105) explained: “The increased distance a dart can be thrown with the atlatl is a function of the increased mechanical advantage which the atlatl provides by increasing the length of the moment arm.” Atlatls therefore represent an amplification of endosomatic energy to apply force.

The mechanical principles of bow hunting on the other hand, requires both the understanding of directional force transmission through launching a small, sharply tipped weapon (the arrow), as well as the understanding that the stored energy in a bent branch, similar to that of some ethno-historically used spring traps [also see Wadley (2010) for archeological evidence], can be used to propel such a projectile forward (Lombard and Phillipson, 2010). The use of such exosomatic energy storage (i.e., energy generated and stored outside of the human body), and the subsequent fast release of elastic energy is the distinctive engineering principle of bow-and-arrow weaponry (Carignani, 2016).

Bow hunting is a clear example of how at least two domain-specific causal node sets (i.e., the engineering principle of leverage and the principle of exosomatic energy-storage) are being brought together to form a single inter-domain causal network (a hunting machine). We therefore argue that the causal understanding involved in bow hunting indicates minds that are able to apply abstract engineering concepts across different knowledge domains. Thus, evidence for the use of such a technology in the deep past reflects the basic principles of causal network understanding of grade 7. We suggest, however, that within each grade of causal understanding there is variation in levels of complexity.

### Forces Acting Over Time

A more complex form of causal network reasoning about a ‘force’ that operates at an extended period of time and maybe across a long distance, and often out of the sight of the hunter, is the use of poisoned arrows where animals are wounded, but often tracked for many hours or even days before finally killed and harvested (see Bradfield et al., 2015). Poison is not a physical force, rather it functions chemically, adding yet another domain-specific node set to that of bow hunting. When preparing and using a poisoned arrow, the hunter must rely on more advanced forms of reasoning and planning than for the other technologies discussed here. Thus the use of poisoned arrows is clearly an example of advanced grade 7 causal thinking.

Ethno-historically recorded use of arrow poisons amongst the Kalahari San indicate that large game such as wildebeest, kudu, oryx, or eland can take up to 12–15 h before they succumb, and often the weakened animals are killed with spears several days after having been shot with poisoned arrows (Silberbauer, 1965). After the animal is hit with an arrow the hunter/s track them over long distances applying speculative tracking as described by Liebenberg (1990, 2013). Previously we have argued that such tracking demonstrates how humans create meaningful causal network hypotheses, i.e., an advanced form of grade 7 causal reasoning [see Lombard and Gärdenfors (2017) for discussion]. This was based on combining different forms of knowledge, including intimate knowledge of kin, non-kin and animal behavior and their inanimate signs, together with knowledge about the landscape (its geographic features, water sources, vegetation, etc.), abstract causal understanding and the mental maps, thought processes and social contexts of the tracker.

Adding poison [often consisting of several ingredients and heat treated (see Bradfield et al., 2015)] to the mix of a bow hunter’s arsenal, implies an understanding that a killing or weakening force can be physically and visibly applied through the poisoned arrow tip, but that the force then works ‘on its own’ and over time to provide the desired effect. The abstraction of understanding that a ‘soft,’ seemingly unforceful chemical substance might deliver lethal force working over an extended period in the absence of the hunters requires advanced causal reasoning. Today’s Kalahari bow hunters, similar to their past counterparts, “may not have a formal understanding of chemistry or chemical reactions, but they have an indigenous knowledge system that enables them to use plant and animal extracts effectively for medicines or poisons” (Bradfield et al., 2015, p. 39).

Wadley (2010) has argued that out-of-sight, long-distance action involving response inhibition, such as setting snares, seems to be a convincing proxy for complex cognition. In line with our argument, Bradfield et al. (2015, p. 39), however, suggested that “the combinations of active and passive meat-getting strategies, and the presence of visible and invisible stages of the hunt, make the use of poisoned arrows a more complex behavior than either snaring or bow hunting alone.” Here we suggest that bow hunting with poisoned arrows represents a deep network of reasoning similar in complexity to the modern human mind of today. Currently, it is thought that poisoned bone-tipped arrows could have been used from about 43,000 years ago in Southern Africa (e.g., d’Errico et al., 2012; Robbins et al., 2012). Whereas earlier evidence might yet be revealed, we suggest that this age can now be seen as the probable minimum for enhanced or complex levels of grade 7 causal understanding.

The upshot from this study is that we find a strong correlation between age of technology and complexity of causal reasoning. We probably see a gradual development in, and between and within, both grades 6 and 7 causal understanding over the last 500 thousand years of our evolution. If we accept current archeological evidence then the chronology can be reconstructed as follows:

• Hunting with spears from about 500–400 thousand years ago associated with *H. heidelbergensis* and *H. neanderthalensis* (Dennell, 1997; Wilkins et al., 2012).
• Hunting with throwing spears from about 300 thousand years ago associated with *H. sapiens* and *H. neanderthalensis* (Thieme, 1997; Villa and Soriano, 2010, but see Churchill, 1993; Schmitt et al., 2003).
• Hunting with spear-throwers/atlatls and darts from about 100 thousand years ago associated with *H. sapiens* and perhaps *H. neanderthalensis* (Brooks et al., 2006; Shea, 2006).
• Hunting with bows and arrows from about 65 thousand years ago, and thus far exclusive to *H. sapiens* (Backwell et al., 2008; Lombard and Phillipson, 2010).
• Hunting with poisoned arrows at least from about 43 and 24 thousand years ago exclusive to *H. sapiens* (d’Errico et al., 2012; Robbins et al., 2012).

## Discussion

Here we have argued that hominins became efficient tool users and tool inventors because their reasoning about causes was extended across causal networks and into the physical domain, and that they became increasingly skilled at understanding causation at a distance in space and through time. Advanced levels of grade 7 causal network understanding, represented here by hunting machines and chemical substances operating over long distances or remotely, are similar to concepts of fluid intelligence (e.g., Carroll, 1993) and analogical reasoning (Green et al., 2010). It also reminds of Mithen’s (1994, 1996) notion that only when his four ‘domains of intuitive intelligence’ (i.e., linguistic, social, technical, and natural history) are fully integrated, we are able to generalize abstract knowledge from one domain to others into the creative, innovative and flexible solutions characteristic of modern humans. He saw evidence for such advanced levels in cognitive fluidity from about 60 thousand years ago in the archeological record (see Haidle, 2010 for further discussion), at roughly the same time we start seeing archeological evidence for bow hunting in Africa (Lombard, 2016).

We have used hunting weaponry as an example to illustrate the interplay between more advanced causal thinking concerning force dynamics and technological development. The weaponry successively allowed action over increasing space and time, and ultimately also exploited stored energy and chemical compounds to extend the range of applied forces. If our model for the evolution of causal understanding is robust, there should be cognitive and neuroscientific correlates to support it. It is beyond the scope of this contribution to provide an exhaustive review on such literature, but below we highlight some research to this effect.

A cognitive archeological study demonstrated that, compared to spear hunting, conceptual, technological, and behavioral modularization and flexibility is amplified during bow hunting (Lombard and Haidle, 2012; also see e.g., Carignani, 2016). This enables an almost endless variety of element combinations in operational chains to reach single or multiple goals, offering the instantaneous and spontaneous capability to effectively handle any one possibility or situation out of a suite of diverse (foreseen and unforeseen) scenarios (Lombard and Haidle, 2012). It allows for a range of cognitive and cultural complexity and flexibility, basic to human behavior today, applied in the most complex of technologies (Lombard, 2016). This is consistent with our grade 7 causal network understanding, and with the archeological record where spear hunting (whether thrust or thrown) precedes bow hunting by hundreds of thousands of years.

In the only neuro-archeological experiment thus far conducted on spear throwing vs. arrow shooting, results indicated that some elements of the central executive were probably in place for Stone Age spear hunters (Williams et al., 2014). This interpretation suggests that spear-hunting Neanderthals and *H. heidelbergensis* possessed some forms of executive functioning, and we therefore argue that they have evolved at least some (in the case of *H. heidelbergensis*), if not all (in the case of Neanderthals) of the capacity for grade 6 causal reasoning. Shooting arrows with a bow, on the other hand, showed statistically significant higher levels of neural activity compared with spear-throwing. Arrow-shooting seems to require increased visual acuity, context updating, internal attention, mental rehearsal, sustained attention and memory load (Williams et al., 2014). Today, these are seen as uniquely human traits, enabling us to synchronize ideas or concepts with motor planning and task execution (Ayres, 1985; May-Benson and Cermak, 2007), which is consistent with our grade 7 causal understanding.

A synthesis of neuro-scientific work on the cognitive processes that give rise to human causal (relational) reasoning indicates that prefrontal areas show domain independence during high-level causal reasoning, while areas within the temporal, parietal, and occipital lobes exhibit evidence of domain dependence in reasoning (e.g., Krawczyk, 2012). Additionally, neuropsychological and neuroimaging studies, indicate that human behaviors associated with complex tool use is facilitated by functionally specialized networks involving temporal, parietal and frontal areas (Johnson-Frey, 2004). Goldenberg and Spatt (2009) found that parietal lesions impaired mechanical problem solving (i.e., understanding how one tool interacts with other tools, which is essential for conceptualizing and using a ‘machine’ such as a bow and arrow). In sum, a shared and integrated fronto-parietal network is key in human spatial attention, space-related behaviors and fluid reasoning/intelligence (e.g., Doricchi et al., 2008; Hampshire et al., 2011; Barbey et al., 2012).

Whereas archaic and modern human frontal skull bones are known to be externally distinct, CT scans of mid-Pleistocene, Neanderthal and modern human crania revealed that the internal prefrontal structure showed no significant alteration over a period of about 500 thousand years of human evolution (Bookstein et al., 1999). This is consistent with the neuro-archeological work that found evidence for some elements of the central executive in spear hunting (Williams et al., 2014), an activity strongly associated with Neanderthals, and our interpretation that they had grade 6 causal understanding.

Bruner’s (2010) palaeo-neurological analysis, however, shows that of all hominins only *H. sapiens* displays a general enlargement of the complete parietal surface, and that the variation in morphological details of that region suggests neuro-functional differences in visuospatial integration such as the “recognition and codification of the outer spatial environment and the associated integration between the outer frame and the inner perceptions” (Bruner, 2010, p. S77). We argue that effective fronto-parietal integration, as is observed in normal modern humans today, is only possible after this evolutionary development. It is also a critical development for the human ability to hypothesize about technological solutions for problems functioning with extended space-time dimensions, such as hunting with a snare or a poisoned arrow.

With bow hunting, reasoning and problem solving depend on the ability to represent and integrate complex relationships (e.g., Kroger et al., 2002). Causal/relational complexity increases with the number of interdependent elements across space and through time that must be simultaneously considered to solve a problem. Hunting remotely in the ‘mind’s eye’ with poison or snares represents high-level grade 7 causal understanding (relational reasoning) with a delayed spatiotemporal aspect and a highly developed ability to visualize, imagine or pre-empt an outcome. The ability to imagine a range of potential outcomes, to understand the consequences of those outcomes, and to grasp that the outcomes may have been different if any of the preceding circumstances varied (i.e., counterfactual thinking, as in Baird and Fugelsang, 2004) is key to current modern human reasoning.

It seems that the neural hardware for effective counterfactual reasoning only reach its final development relatively late in humans – after adolescence (Baird and Fugelsang, 2004). Functional brain imaging on healthy 8–19 year olds also suggests that neuro-maturational changes associated with visuo-spatial relational reasoning shift from a widespread frontal pattern in childhood to predominant parieto-frontal activation in late adolescence (Eslinger et al., 2009). Together with the relative late enlargement of the complete parietal surface in hominin evolution (Bruner, 2010), these observations may explain why bow hunting is unique to *H. sapiens*, and why space-time-delayed hunting with poisoned arrows appear so late in our technological repertoire.

## Conclusion

The main contribution of this article is to place emphasis on the role of force dynamics to understand the evolution of causal cognition. This supports Malafouris’ (2013) position that there is a back-and-forth interaction between the extension of human body through technology and the extension of our cognitive abilities. It is also in line with Bruner and Lozanos’ (2014) argument that the development of the human mind cannot be decoupled from the use of technology. Ultimately, if scenarios about our cognitive evolution are to be robust, it is important that multiple lines of evidence come together to mutually strengthen and/or constrain our interpretations.

We see a chronological progression, starting from thrusting spears, through bow hunting with poisoned arrows that maps onto our progression of causal thinking, and potentially onto the evolution of the Neanderthals and *H. sapiens* from the more archaic *H. heidelbergensis*. Our examples of weaponry are not exhaustive. We chose them to illustrate the connections of technologies to different grades of causal reasoning and to highlight potential developments within grades. Our analytic tools not limited to hunting technologies, but could be extended to other technologies such as thread-based material culture from early twine production to complex knotting (e.g., knitting) and weaving with machines.

The theory of causal grades can explain why spear hunting is older than bow hunting since the causal reasoning required for using spears is less advanced than that for using bows. We suggest that clear examples of grade 7 causal thinking are only found relatively late. Currently, it seems that from about 70 thousand years ago, technologies such as bow hunting and using spring traps rely on using and understanding exosomatically stored energy. Thus far it seems that such technologies are unique to *H. sapiens* and may reflect variation in causal understanding between modern and archaic groups. Poisoned arrows could have been used from about 43 thousand years ago (Robbins et al., 2012), and arrow poison itself preserved on a wooden applicator dated to 29 thousand years ago (d’Errico et al., 2012). Currently, these finds represent the earliest evidence for hunting behavior that requires enhanced grades of causal cognition, spanning several knowledge domains. This interpretation implies continued intra-species cognitive evolution for humans in line with what was suggested by, for example Malafouris (2010) and Kolb and Gibb (2011).

## Author Contributions

Both authors have made a substantial, direct and intellectual contribution to the work, and approved it for publication.

## Conflict of Interest Statement

The authors declare that the research was conducted in the absence of any commercial or financial relationships that could be construed as a potential conflict of interest.
